# Analysis of immune-related genes and potential therapeutic drugs in refractory lupus nephritis: Comparison with drug-sensitive lupus nephritis based on RNA transcription data

**DOI:** 10.1097/MD.0000000000046120

**Published:** 2025-11-28

**Authors:** Bo Shao, Kaixiu Wu, Li Xiao, Yanggen Zuo, Shui Wan, Jinbo Pi, Zhengkai Fan, Zhongxiong Han, Pingping Sun

**Affiliations:** a Zhaotong Hospital of Traditional Chinese Medicine, Urinary Surgery, Zhaotong City, Yunnan Province, China; b Dali University, Dali City, Yunnan Province, China; c Wuhu Hospital of Traditional Chinese Medicine, Wuhu City, Anhui Province, China.

**Keywords:** GEO database, immune core genes, potential therapeutic drugs, RLN

## Abstract

Based on the gene expression omnibus database, the immune-related genes associated with refractory lupus nephritis (RLN) are analyzed by comparing the RNA expression data of drug-sensitive lupus nephritis (DLN) with that of RLN. Subsequently, potential therapeutic drugs were screened based on the identified genes. The RNA expression datasets related to DLN and RLN were sourced from the gene expression omnibus database. Differential analysis was performed to ascertain differentially expressed genes. These differentially expressed genes were then cross-referenced with immune-related genes obtained from the ImmPort database to identify potential candidate genes. To investigate the underlying biological mechanisms, gene ontology and kyoto encyclopedia of genes and genomes analyses were performed. Core genes were subsequently identified using the STRING database, along with least absolute shrinkage and selection operator regression analysis and support vector machine-recursive feature elimination (SVM-RFE) techniques. The receiver operating characteristic curve was utilized to confirm and assess the significance of the core genes. A nomogram prediction model was developed, with the concordance index (C-index) applied to evaluate its precision. The immune cell infiltration in both RLN and DLN was examined, investigating the relationships between core genes and immune cells, as well as interactions among the immune cells. Coremine Medical was engaged to discover traditional Chinese medicines that exhibited significant associations with the core genes. Additionally, the Traditional Chinese Medicine Systems Pharmacology database was employed to identify small molecule compounds with therapeutic potential found within these traditional medicines. Finally, the protein structures of the key genes and their corresponding small molecule compounds were processed using Vina software and PyMol software for molecular docking, ensuring the reliability of the results. In this investigation, a total of 5 essential genes were pinpointed: MMP-9, IRF7, CCR6, OAS1, and IFIH1. Furthermore, small molecule compounds that may have therapeutic potential were identified based on these central genes, including tanshinone IIA (Tan IIA), luteolin, ellagic acid, baicalein, and quercetin. The immune mechanism of RLN is complex, arising from a synergy of multiple factors, targets, and pathways. This approach is valuable for elucidating the mechanisms underlying RLN resistance and offers a novel perspective for enhancing immunotherapy effectively.

## 1. Introduction

LN is an immune complex nephritis primarily associated with systemic lupus erythematosus (SLE) that affects the kidneys. It is characterized by proteinuria, active urinary sediment, hypertension, renal insufficiency, and various complications. LN represents a significant complication of SLE.^[1]^ Currently, the primary treatment for LN involves the use of immunosuppressive therapies.^[2]^ The advancements in traditional cyclophosphamide (CTX) therapy, along with the introduction of mycophenolate mofetil (MMF), tacrolimus (TAC), and novel biological agents such as belimumab, as well as multi-target therapies, have contributed to an increase in the remission rate of LN induction therapy, which now ranges from 52.1% to 83.5%.^[3]^ Active immunosuppressive therapy in LN patients can extend the duration before further kidney deterioration occurs; however, it is important to note that 14% to 33% of patients remain unresponsive to first-line treatments, experience intolerance, or suffer from rapid recurrence following initial remission, ultimately progressing to refractory lupus nephritis (RLN). This situation can lead to continuous renal function decline and may progress to end-stage renal disease.^[4]^ In light of the aforementioned situation, it is imperative to identify novel treatment strategies for patients with RLN involvement. Utilizing the gene expression omnibus (GEO) database, this study examined the differences in IRGs between DLN and RLN. The research focused on identifying key genes, elucidating the mechanisms underlying drug resistance in RLN, and screening small molecule compounds as potential therapeutic agents based on these core genes. This approach aims to provide clinical insights into the mechanisms of drug resistance and facilitate the development of new therapeutic agents.

## 2. Material and methods

### 2.1. Data download and pre-processing

Acquire the transcriptome dataset labeled GSE224705 from the GEO database (https://www.ncbi.nlm.nih.gov/geo/) as of June 23, 2024. This dataset includes 300 samples from the DLN (Control group) and 128 samples from the RLN (Treatment group) for the extraction and analysis of RNA data. Furthermore, retrieve 1793 IRGs from the ImmPort database (https://www.immport.org/). All resources utilized in this research are derived from publicly accessible data, thus eliminating the need for patient informed consent. The R4.4.1 software was employed to standardize the data using the Min-Max normalization method, scaling the data to the interval [0, 1]. The same version of R software was utilized for all subsequent operations.

### 2.2. Screening of intersection genes

Based on the gene annotation information from the GPL13158-5065 platform, gene IDs were transformed into gene names. For genes represented by multiple IDs, the most significantly differentially expressed value was selected. The analysis was performed using the “limma” package in R software, with filtering parameters set to |logFC| >1 and a *P*-value threshold of <.05. An examination of differential expression was carried out on the gene expression datasets from the DLN and RLN, aimed at identifying genes that show significant differentially expressed genes (DEGs). Furthermore, volcano plots and heat maps depicting these DEGs were created. To uncover shared genes, an overlap analysis between DEGs and IRGs was conducted, followed by the construction of a Venn diagram to visually represent these intersections.

### 2.3. KEGG and GO enrichment analysis

The files c2.cp.keg.v7.5.1.symbols.gmt and c5.go.v7.5.1.symbols.gmt from the MSigDB database were obtained for the purpose of conducting functional gene set analyses related to gene ontology (GO) and kyoto encyclopedia of genes and genomes enrichment. A total of 1000 random combinations were configured, with a false discovery rate (FDR) of 1 and a significance level of *P* <.05.

### 2.4. Screening core genes

The STRING database was utilized to set the parameter “medium confidence = 0.400” for analyzing the interactions between intersecting genes and constructing the protein interaction network. Following this, the ten candidate genes exhibiting the highest “degree” values were determined with the aid of the CytoHubba plug-in. Additionally, the co-expression relationships between these selected candidate genes were analyzed in greater depth utilizing the “corrr” package within R software.

Least absolute shrinkage and selection operator (LASSO) regression analysis and the SVM-RFE algorithm were employed to further screen candidate genes, utilizing the “glmnet” and “e1071” packages in R software. We set the key parameter, alpha, of the LASSO regression analysis to 1, performed 10-fold cross-validation for the SVM-RFE algorithm, and established a feature elimination threshold of 50, specifically “k = 10, halve.above = 50.” The results from both methods were then intersected to identify the core genes. Additionally, we downloaded the gene position information file from Ensembl (https://ftp.ensembl.org/pub/) to analyze the chromosomal positions of the core genes.

### 2.5. Validation of core genes

The “ggpubr” package in R was employed to create the box plot for analyzing the expression variations of core genes in DLN and RLN. Subsequently, the “pROC” package was used to construct a receiver operating characteristic curve to evaluate the diagnostic capabilities of these core genes. In addition, a nomogram model for the core genes was established using the “rms” package, which was then validated via a consistency calibration curve.

### 2.6. Immune infiltration analysis

The corrected gene expression data were utilized for immune infiltration analysis using the CIBERSORT algorithm in R. A filtering condition of “*P*-value <.05” was established, and a comparative analysis was conducted to assess the differences in the relative content of immune cells between the Treatment group and the Control group. The R function “p.adjust” was employed for FDR correction, with the FDR threshold set at 0.05. The analysis incorporated the “limma,” “tidyverse,” “dplyr,” “ggplot2,” and “linKET” packages in R to evaluate the correlations among core genes, immune cells, and between different immune cell types.

### 2.7. Potential drug prediction and molecular docking of key genes

The Coremine Medical (CM) platform (https://coremine.com/medical/) integrates a vast array of interaction data among chemicals, genes, functional phenotypes, and diseases, thereby offering significant convenience for investigating the potential mechanisms of drug action. Core genes were imported into the database to facilitate the search for potential Chinese medicines. The parameters of “OB (oral bioavailability) ≥30%” and “DL (drug-likeness) >0.18” were set using the traditional Chinese medicine systems pharmacology (TCMSP) database (https://www.tcmsp-e.com/) to identify small molecule compounds with possible drug-like characteristics. The structures of these compounds were retrieved from the PubChem database (https://pubchem.ncbi.nlm.nih.gov/), while the protein structures corresponding to the key genes were obtained from the RCSB PDB (https://www.rcsb.org/). Subsequently, we imported the protein structure of the core gene and the corresponding small molecule compound structure into AutoDock Vina 1.5.6 and PyMOL 3.0 for molecular docking analysis, subsequently calculating the free energy.

## 3. Results

### 3.1. Intersection genes of DEGs and IRGs

**Figure 1.**
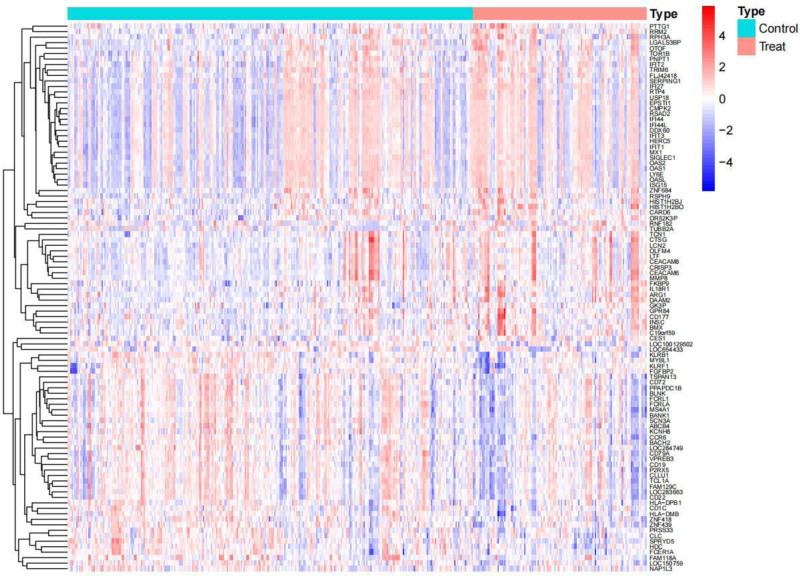
Heatmap of differentially expressed genes. In this representation, red indicates up-regulation of genes, while blue signifies down-regulation. The intensity of the color corresponds to the level of significance, with darker hues indicating greater significance and lighter hues indicating lesser significance.

**Figure 2.**
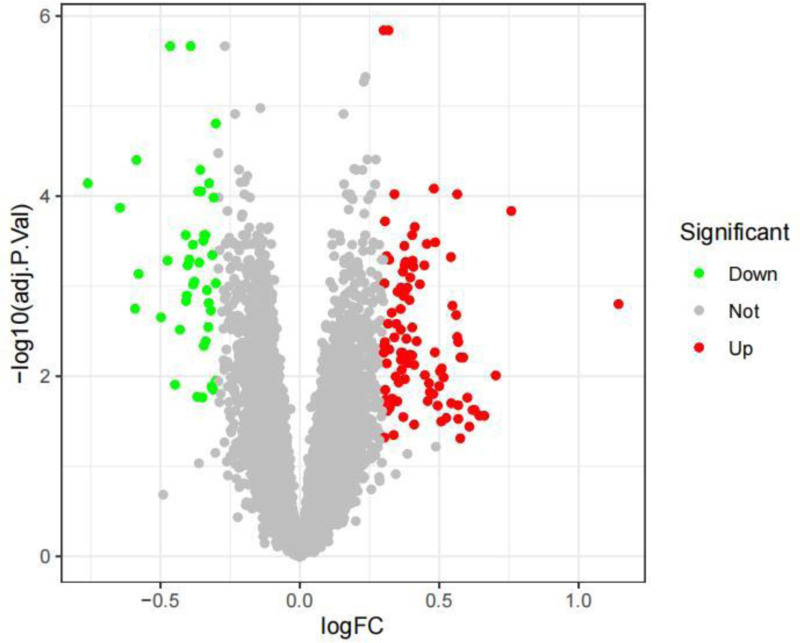
Volcano map illustrating differentially expressed genes, where red signifies up-regulation of gene expression and green indicates down-regulation.

**Figure 3.**
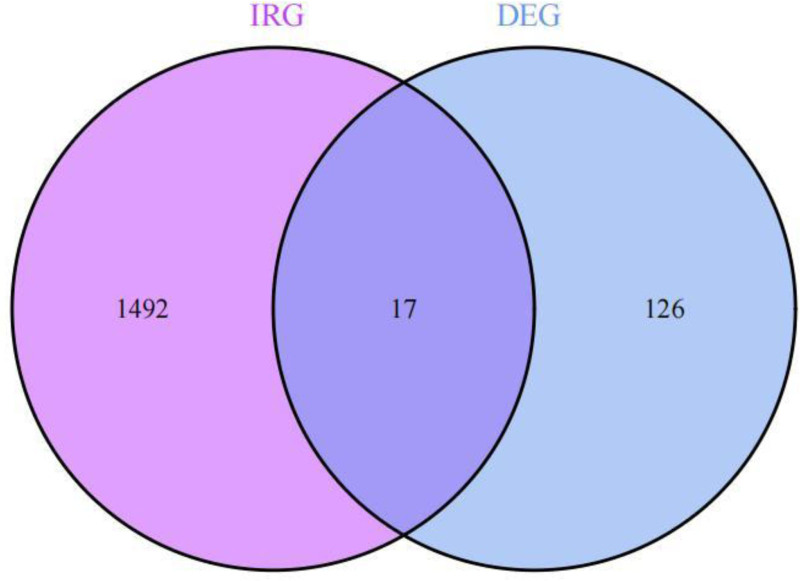
Venn diagram depicting the relationship between differentially expressed genes and immune-related genes.

Based on the defined screening parameters of |(logFC)| >1 and *P*-value <.05, a total of 143 DEGs were detected within the dataset designated GSE224705 (Figs. 1 and 2). The overlap of these 143 DEGs with 1793 IRGs sourced from the Import database led to the discovery of 17 intersecting genes (Fig. 3).

### 3.2. Kyoto encyclopedia of genes and genomes and GO enrichment analysis

**Figure 4.**
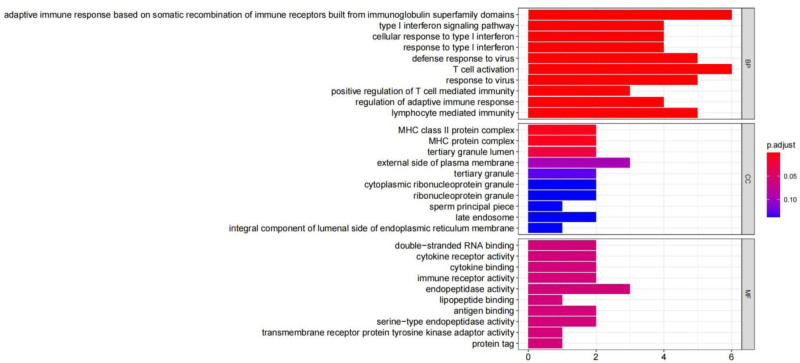
GO enrichment results are presented in a bar chart format, illustrating ten representative BP, CC, and MF. The abscissa denotes the number of gene enrichments, while the ordinate indicates the enrichment pathways. A deeper red color corresponds to a smaller *P*-value, whereas a deeper blue color signifies a larger *P*-value. BP = biological processes, CC = cellular components, GO = gene ontology, MF = molecular functions.

**Figure 5.**
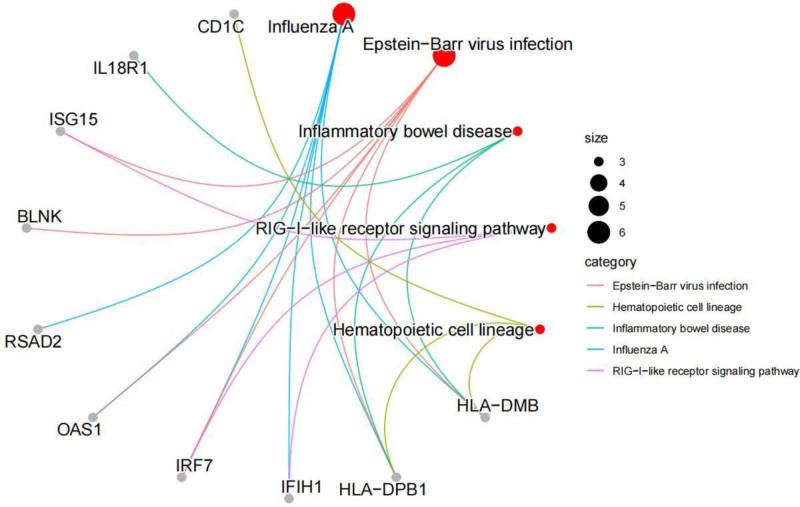
KEGG enrichment result diagram. The lines of various colors represent different signaling pathways. The red circle denotes the pathway, while the gray circle indicates the gene. The size of the red circle corresponds to the number of enriched genes; thus, a larger red circle area signifies a greater number of enriched genes. KEGG = kyoto encyclopedia of genes and genomes.

The enrichment analysis of Gene Ontology suggested that the biological processes related to RLN were mainly focused on the “adaptive immune response relying on somatic recombination of immune receptors consisting of immunoglobulin superfamily domains,” along with various other processes, the molecular functions predominantly involved “endopeptidase activity,” while the cellular components (CC) mainly comprised the “MHC class II protein complex” (Fig. 4). Furthermore, the KEGG enrichment analysis indicated that the overlapping genes were involved in signaling pathways, including “Epstein–Barr virus infection” (see Fig. 5).

### 3.3. Screening core genes

**Figure 6.**
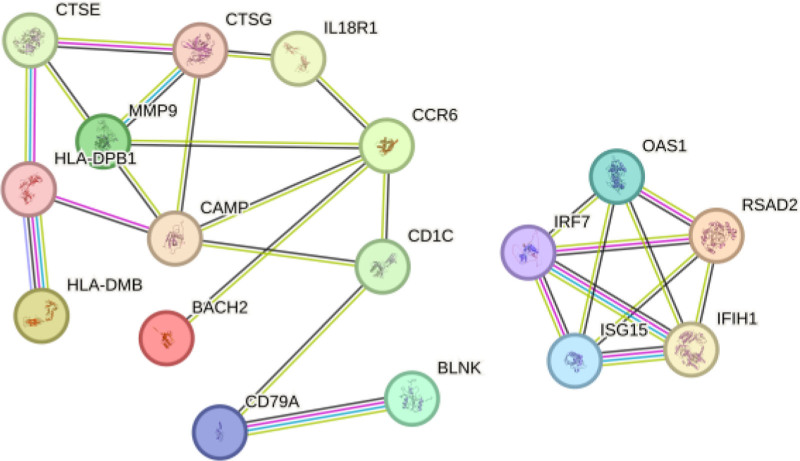
PPI protein interaction network diagram. PPI = protein-protein interaction.

**Figure 7.**
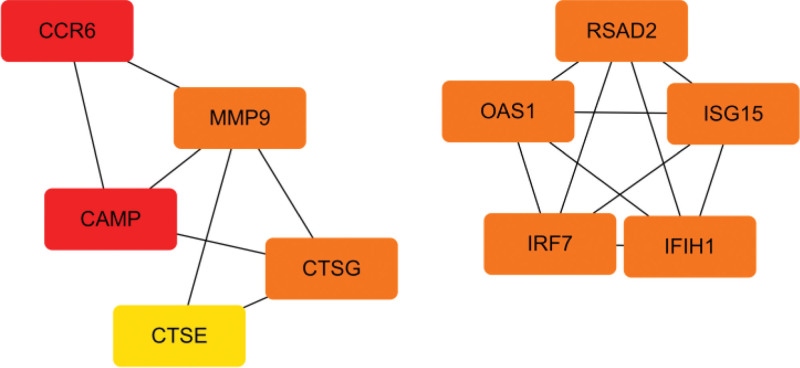
Candidate gene network diagram, where the redder the color, the higher the degree of the gene’s core.

**Figure 8.**
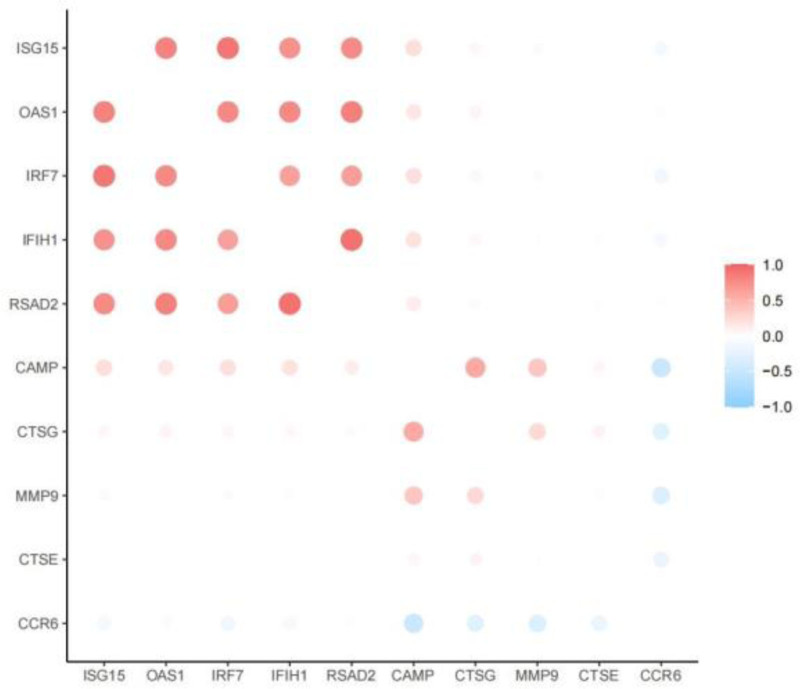
The expression relationship diagram of candidate genes, with red indicating positive correlation and blue indicating negative correlation.

**Figure 9.**
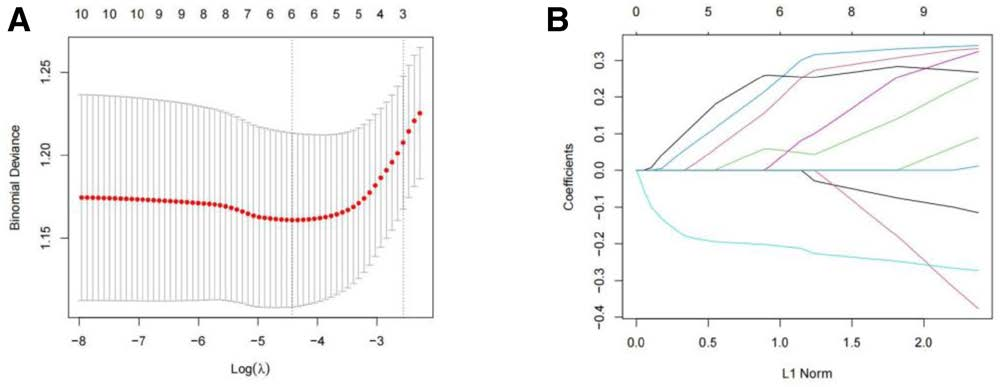
LASSO regression analysis cross-validation diagram (A) and LASSO regression analysis coefficient curve diagram (B). LASSO = least absolute shrinkage and selection operator.

**Figure 10.**
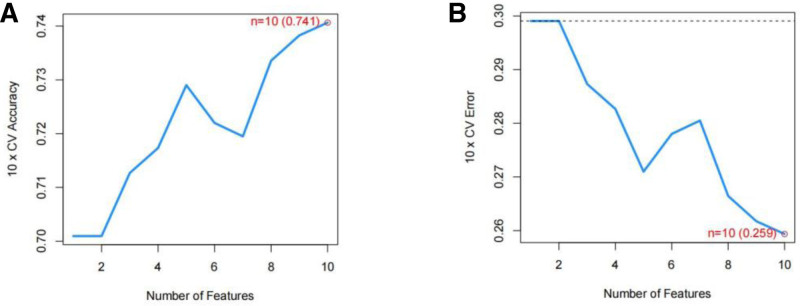
Illustrates the results of the SVM-RFE algorithm. The accuracy graph for SVM-RFE (A) indicates that the highest accuracy is achieved when the curve reaches its peak. Conversely, the error plot for SVM-RFE (B) shows that the error value is minimized when the curve reaches its lowest point. SVM-RFE = support vector machine-recursive feature elimination.

**Figure 11.**
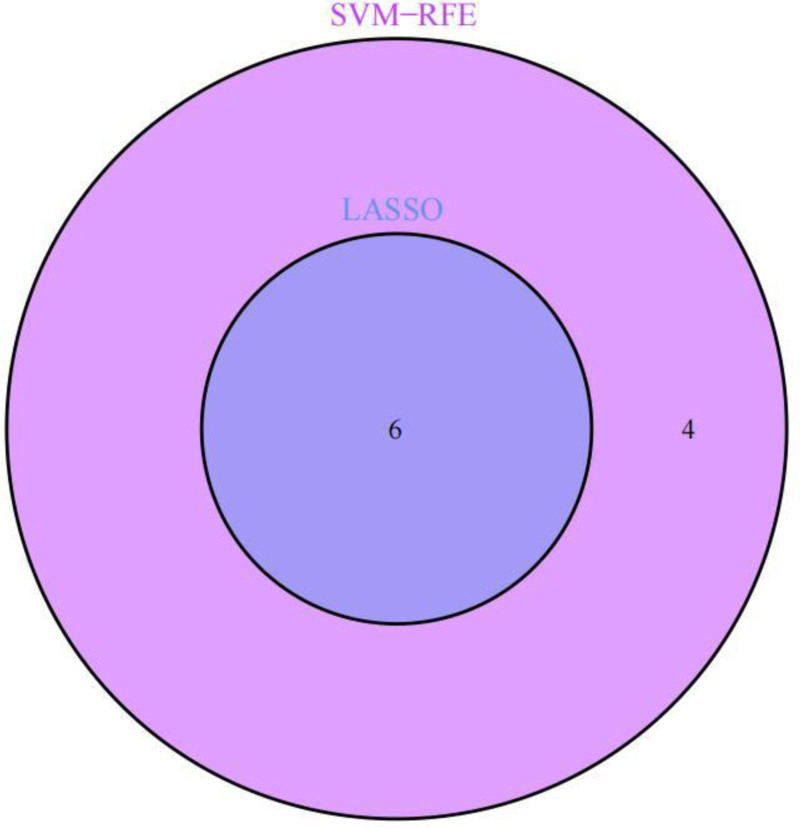
Venn diagram illustrating the intersection of LASSO regression analysis and the SVM-RFE algorithm. LASSO = least absolute shrinkage and selection operator, SVM-RFE = support vector machine-recursive feature elimination.

**Figure 12.**
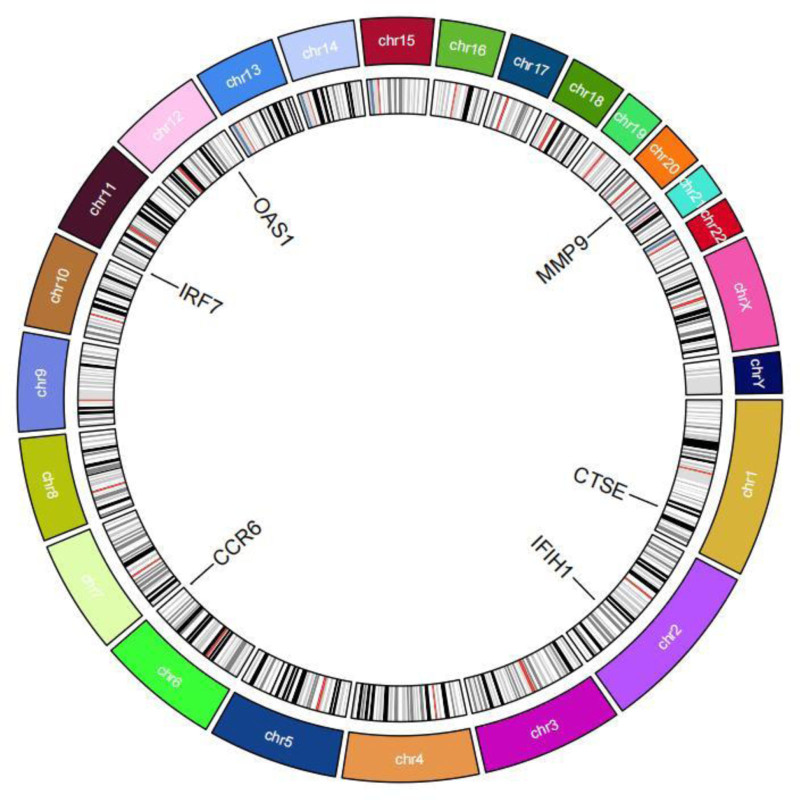
Chromosomal locations of core genes.

The STRING database was employed to examine the relationships between the intersecting genes mentioned earlier, leading to the creation of a protein interaction network diagram (Fig. 6). Following this, the CytoHubba plug-in was used to pinpoint the top 10 candidate genes with the highest “degree” values, which consisted of OAS1, CCR6, MMP-9, IFIH1, IRF7, CTSE, CAMP, CTSG, RSAD2, and ISG15 (Fig. 7). Further analysis of the co-expression relationships among candidate genes revealed a significant negative correlation between CCR6 and the genes CTSE, IRF7, CAMP, and MMP-9, while a significant positive correlation was observed among the remaining genes (Fig. 8). To refine the selection of candidate genes, LASSO regression analysis and the SVM-RFE algorithm were employed. The LASSO regression analysis identified 6 core genes (Fig. 9A and B), whereas the SVM-RFE algorithm identified ten core genes (Fig. 10A and B). The intersection of these 2 analyses yielded the core genes associated with RLN: MMP-9, IFIH1, IRF7, CCR6, OAS1, and CTSE (Fig. 11). Additionally, the positional relationships of the core genes on the chromosome were determined (Fig. 12).

### 3.4. Validation of core genes

**Figure 13.**
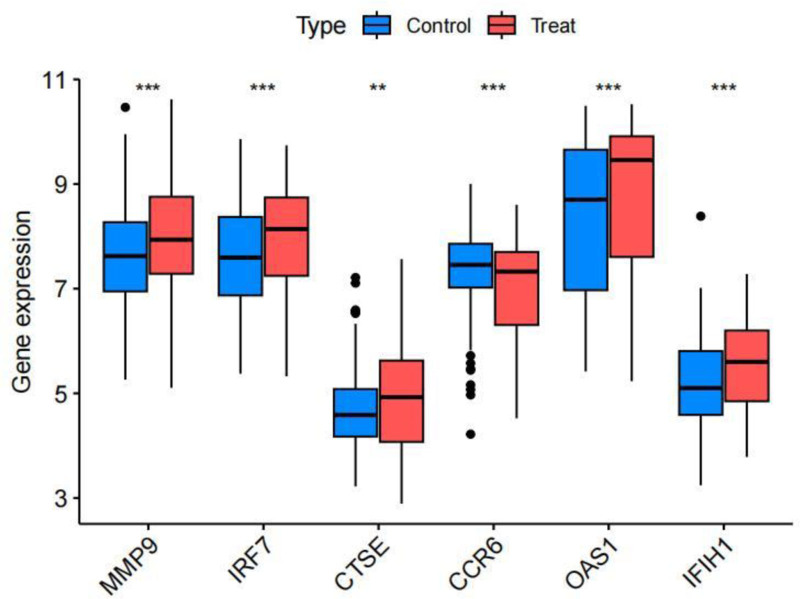
Differential expression levels of core genes, with significance indicated as follows: *: *Ρ*<.05;**: *Ρ*<.001; ***: *Ρ*<.0001.

**Figure 14.**
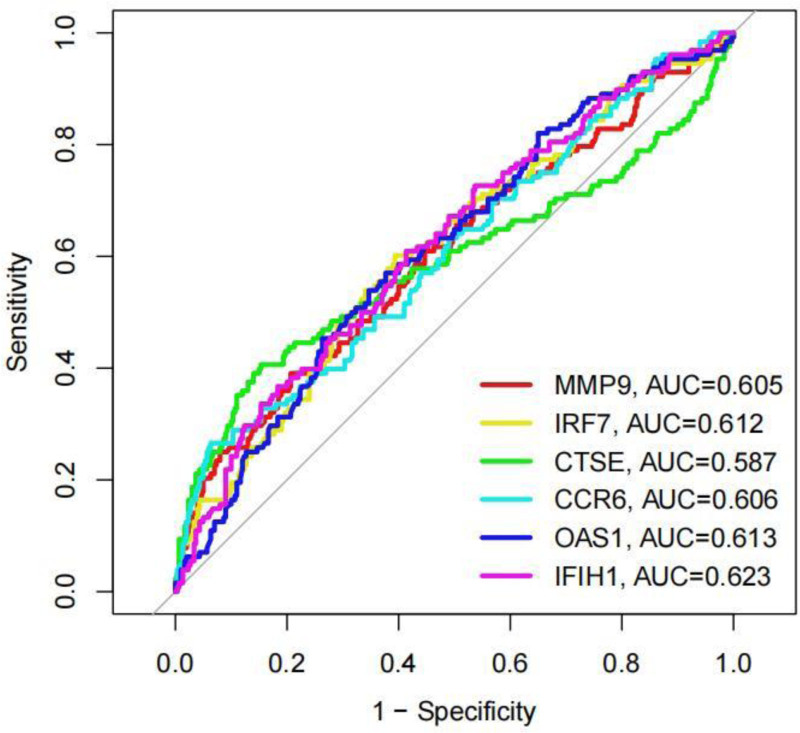
ROC curve analysis of core genes. ROC = receiver operating characteristic.

**Figure 15.**
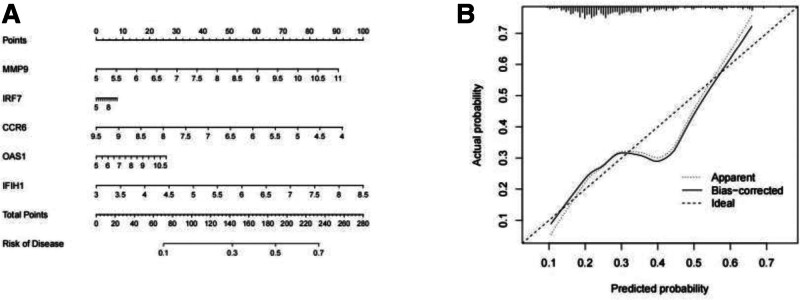
Nomogram (A) and consistency calibration curve; the closer the curve is to the diagonal, the higher the accuracy (B).

The differential expression of core genes between DLN and RLN was analyzed. The results indicated that, with the exception of CCR6, which exhibited low expression in RLN, all other core genes were highly expressed (Fig. 13). Receiver operating characteristic curve analysis revealed that only MMP-9(AUC = 0.605), IFIH1(AUC = 0.623), IRF7(AUC = 0.612), CCR6(AUC = 0.606), and OAS1(AUC = 0.613) demonstrated strong diagnostic ability. Consequently, these 5 genes were identified as the final core genes (Fig. 14). A nomogram model of core genes was established using the “rms” package (Fig. 15A). This nomogram exclusively incorporates the influence of gene expression data on the disease, and the consistency calibration curve suggested that the model possessed good predictability, C-index = 0.83. (Fig. 15B).

### 3.5. Immune infiltration analysis

**Figure 16.**
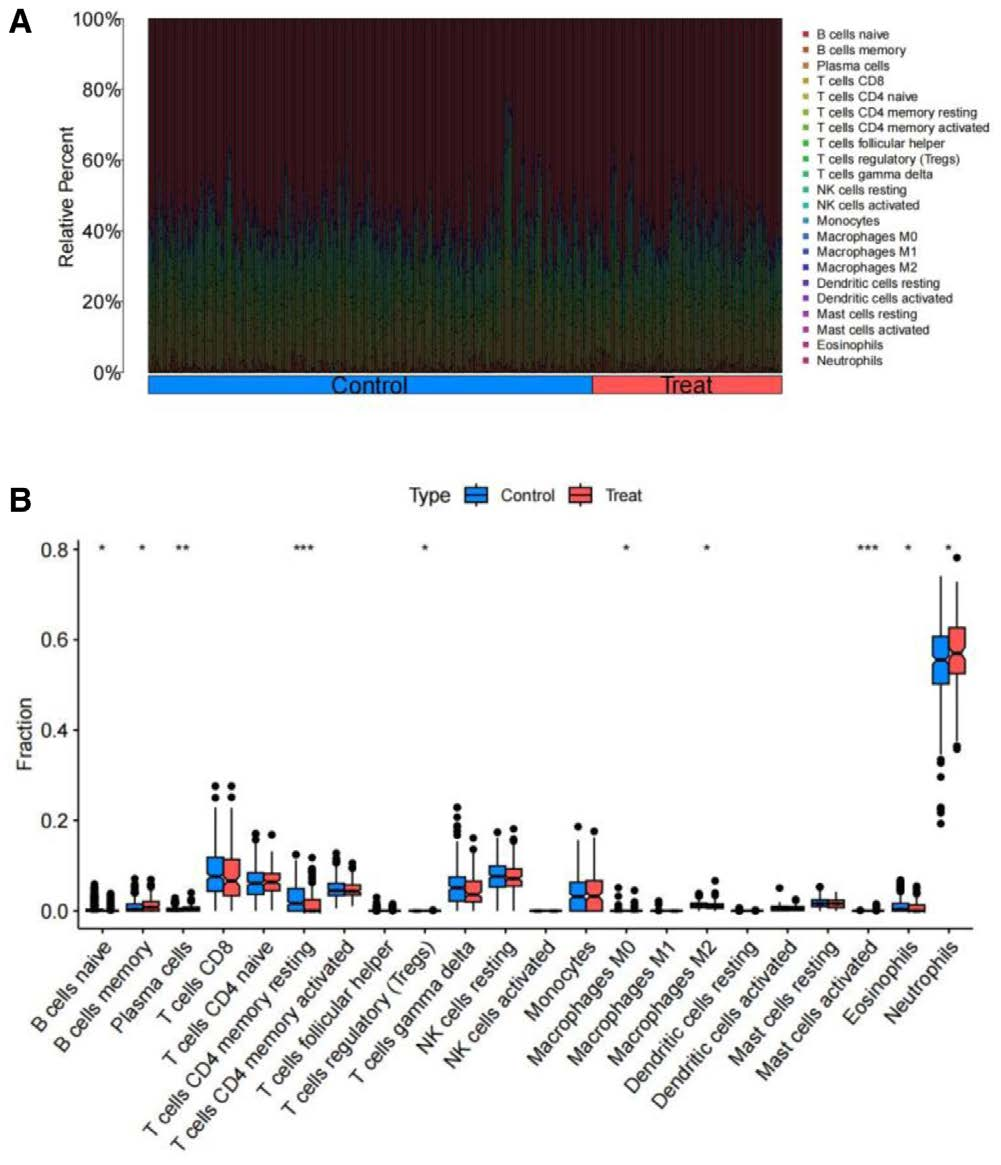
Histogram of immune infiltration analysis (A) and immunoassay box plot, with significance indicated as follows: *: *Ρ*<.05; **: *Ρ*<.001;***: *Ρ*<.0001 (B).

**Figure 17.**
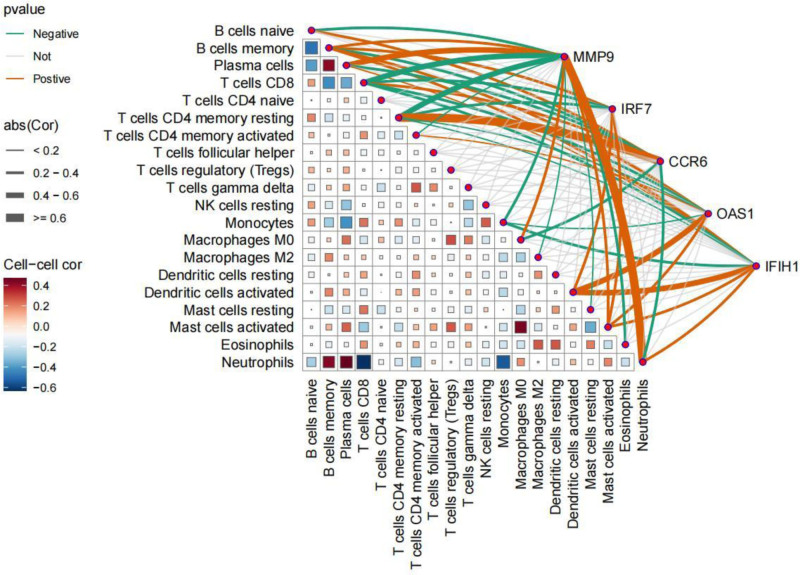
Illustrates the relationships among immune cells, as well as between immune cells and core genes. The triangular area depicts the correlation between immune cell types and their expression levels. Red indicates a positive correlation, while blue signifies a negative correlation; the term “abs” refers to the absolute value of the correlation, with a larger area of “abs” corresponding to a steeper slope of correlation. The connections between core genes and the triangular region are represented by green for negative correlations, orange for positive correlations, and gray for no correlation. Additionally, thicker lines denote stronger correlations.

Through the analysis of immune infiltration with FDR correction, it was observed that the immune cell histogram illustrated the proportion of 22 immune cell types in each sample (Fig. 16A). The adjusted *P*-value was found to be 0.032. At the same time, the box plot of immune cells indicated that the levels of expression for “memory B cells,” “Plasma cells,” “Neutrophils,” and “activated Mast cells” were elevated in RLN patients compared to those in DLN patients. Conversely, “naive B cells,” “resting memory CD4 T cells,” “regulatory T cells (Tregs),” “M0 Macrophages,” “M2 Macrophages,” and “Eosinophils” displayed reduced expression levels in RLN patients when contrasted with DLN patients (Fig. 16B). Furthermore, we discovered a noteworthy correlation between the expression of immune cell types and their relationship with core genes. In particular, CCR6 showed a positive correlation with inactive immune cells, such as “resting memory CD4 T cells,” while demonstrating a negative correlation with active immune cells, like “Neutrophils.” This finding implies that CCR6 may act as an immunosuppressive gene, potentially benefiting the immunosuppressive treatment of RLN. Conversely, the remaining genes exhibit characteristics associated with pro-immune responses (Fig. 17).

### 3.6. Potential drug prediction and molecular docking of core genes

**Figure 18.**
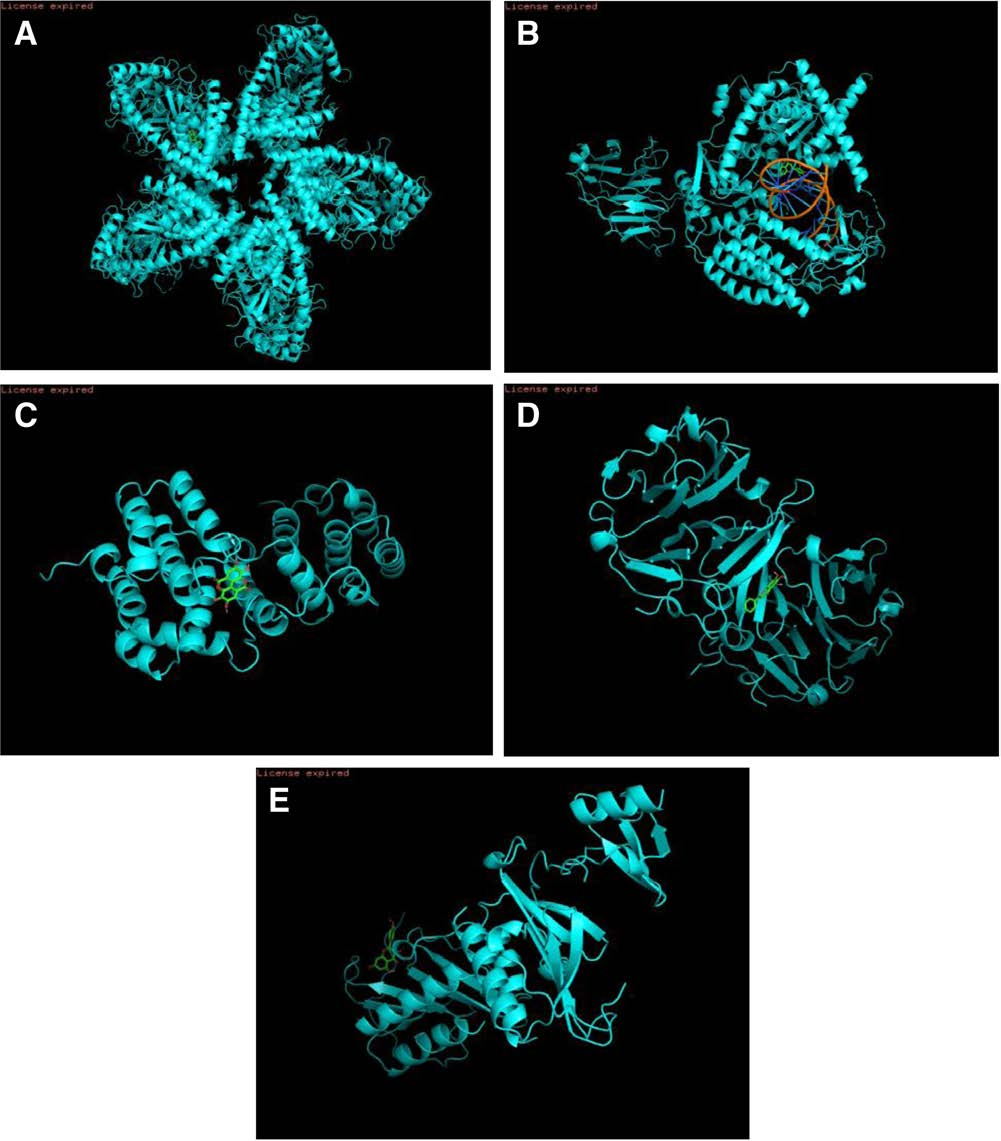
Molecular docking analysis of core genes and small molecule compounds. OAS1 was docked with tanshinone IIA (A), IFIH1 was docked with luteolin (B), IRF7 was docked with ellagic acid (C), MMP-9 was docked with baicalein (D), and CCR6 was docked with quercetin (E).

The core genes were integrated into the CM platform, and ten Chinese medicines, including “Banlangen,” “Chishao,” “Dangshen,” “Daqingye,” and “Huangqin,” were retrieved. The small molecule compounds associated with these Chinese medicines were searched in the TCMSP database, and intersections were identified based on the target genes and key genes of the small molecule compounds. Ultimately, Tan IIA, luteolin, ellagic acid, baicalein, and quercetin were recognized as the most significant small molecule compounds, which were successfully docked with their corresponding key gene proteins. The lowest free energy observed during the docking of OAS1 with Tan IIA is −11.3 kJ/mol (refer to Fig. 18A). For IFIH1 and luteolin, the minimum free energy of docking measures −10.3 kJ/mol (as shown in Fig. 18B). The docking interaction between IRF7 and ellagic acid results in a minimum free energy of −7.9 kJ/mol (illustrated in Fig. 18C). Furthermore, the docking of MMP-9 with baicalein has a minimum free energy of −8.1 kJ/mol (depicted in Fig. 18D). Lastly, CCR6’s docking with quercetin shows a minimum free energy of −8.2 kJ/mol (presented in Fig. 18E). The free energy less than or equal to-7KJ/ mol indicates that the stability is good.

## 4. Discussion

Currently, RLN presents a significant challenge in the treatment of LN. Its resistance to immunosuppressive agents poses an urgent issue that must be addressed, as it critically impacts the life and health of patients. Nonetheless, there is a significant scarcity of effective therapeutic approaches to address both the onset and progression of the disease, as current studies on the mechanisms that contribute to drug resistance in RLN are insufficient. Therefore, this study aimed to identify immune core genes by comparing RLN with DLN, thereby providing preliminary insights into the drug resistance mechanisms of RLN and offering clinical value for its treatment.

### 4.1. Immune core genes

In this research, the data collected from DLN patients acted as the control group to examine the variations in immune gene expression contrasting DLN with RLN. A total of 5 immune core genes were identified and examined, including MMP-9, IRF7, CCR6, OAS1, and IFIH1. Notably, CCR6 demonstrated immunosuppressive efficacy and was classified as a protective gene associated with RLN.

#### 4.1.1. MMP-9

MMP-9 is part of the matrix metalloproteinase family, and its main role is to break down the extracellular matrix (ECM), which is essential for preserving ECM equilibrium.^[5]^ MMP-9 not only directly influences the degradation, synthesis, and transport of ECM components, but it also recruits inflammatory cells to secrete inflammatory factors, mediates inflammation and tissue remodeling, and serves as a key mediator in the process of immune inflammation.^[6]^ Studies have demonstrated that MMP-9 can mediate the inflammatory response through the Wnt5a/MMP-9 signaling pathway, thereby activating the immune response and causing tissue damage.^[7]^

#### 4.1.2. IRF7

IRF7 is a member of the protein family encoded by the interferon (IFN) regulatory factor gene. It features a classic helix-turn-helix structure. Its most significant biological function is to induce the production of type I IFN and participate in the body’s antiviral and immune responses.^[8]^ IRF7 is implicated in the occurrence and progression of various diseases, including autoimmune diseases,^[9]^ respiratory inflammation,^[10]^ and diabetes.^[11]^ Additionally, IRF7 plays a crucial role in renal immune injury. In chronic kidney disease, increased circulating RNA acts as an endogenous damage molecule, significantly elevating the levels of NF-κB, IRF3, and IRF7, thereby regulating the immune inflammatory response associated with chronic kidney disease.^[12]^ In the 5/6 nephrectomy rat model, the expression level of IRF7 was notably increased at 15 and 60 days post-nephrectomy, which may lead to the release of inflammatory factors and promote the formation of the inflammasome by activating the TLR2/4/5-MyD88-TRAF6-IRF7 signaling pathway.^[13]^ This study’s bioinformatics analysis revealed that the expression level of IRF7 was significantly elevated in RLN patients, positioning it at the core of the immune response network, suggesting a close relationship between IRF7 and immune injury.

#### 4.1.3. CCR6

CCR6 belongs to the G protein-coupled 7 transmembrane protein receptor family and serves as the receptor for CC chemokine ligand 20 (CCL20). CCR6 has been demonstrated to be expressed in various leukocyte subsets, including T cells, dendritic cells (DC), and natural killer cells.^[14]^ Studies indicate that CCL20 mediates the expansion and activation of the RORγt + T regulatory cell population through CCR6 signaling, which is associated with the Th17-specific transcription factor RORγt. These RORγt + Tregs possess the capacity to enhance immunosuppression.^[15]^ The level of CCR6 + ILC3 is positively correlated with the levels of granulocyte-macrophage colony-stimulating factor (GM-CSF) and type 1 conventional DC. The latter is also associated with a decline in renal function and a reduction in ILC3, particularly the decrease in CCR6 + ILC3, which is linked to the deterioration of renal function.^[16]^ This study demonstrates that CCR6 plays an immunosuppressive role in RLN patients, which may help mitigate renal damage, aligning with the aforementioned research findings.

#### 4.1.4. OAS1

OAS1 belongs to the OAS protein family, which includes 4 genes responsible for encoding active OAS enzymes: OAS1, OAS2, OAS3, and OASL. These enzymes are essential for innate immunity. Acting as an interferon-stimulated gene, OAS1 facilitates the enhancement of the interferon transcription program, boosts the activity of natural killer cells, macrophages, and T lymphocytes, and manages the immune response of the body. Studies have shown that different variants of OAS1 are associated with inflammatory diseases. These variants have the ability to activate RNase L even without stimulation from double-stranded RNA, leading to RNA cleavage, changes in the transcriptome, and inhibition of translation.^[17]^ Research by Thomas M^[18]^ highlighted that the mRNA expression levels of OAS1, OAS2, and OASL among patients with SLE were significantly elevated compared to those in the normal control group, suggesting a role for OAS isoenzymes in the autoimmune mechanisms related to SLE. Additionally, Landolt-Marticorena^[19]^ observed that the levels of OAS1 and B cell activation factor (BAFF) in the peripheral blood of SLE patients were increased, establishing a correlation between these 2 factors. Moreover, the abnormal proliferation of B cells in the peripheral blood of SLE patients, in comparison to the control group, implies that the heightened expression of OAS1 might be associated with this atypical B cell proliferation.

#### 4.1.5. IFIH1

IFIH1 is a type I interferon-induced gene (IFIG) that encodes the melanoma differentiation associated gene 5 (MDA5). Munroe^[20]^ found that the IFIH1 gene is closely related to the expression of interleukin-6 (IL-6), interferon-induced protein 10 (IP-10), and autoantibodies. This suggests that IFIH1 may play a role in the pathogenesis of SLE by regulating the inflammatory response. Additionally, mice with the IFIH1 G821S missense mutation exhibited lupus-like symptoms. The researchers detected the presence of antinuclear antibodies and anti-dsDNA antibodies in the serum of these mice and observed the deposition of immunoglobulins and complements in kidney tissues. Furthermore, inflammatory cytokines and chemokines, including IFNβ, IL-6, and CXC chemokine ligand 10 (CXCL10), were significantly upregulated in the kidneys. This experiment indicates that IFIH1 may trigger the body’s autoimmune response by activating the IFN-I pathway.^[21]^

### 4.2. Small molecule compounds

In this study, immune core genes were identified as therapeutic targets, and 5 small molecule compounds with potential therapeutic effects were screened using TCMSP and other databases. These compounds include: Tan IIA, luteolin, ellagic acid, baicalein, and quercetin.

#### 4.2.1. Tan IIA

Tan IIA, a compound belonging to the class of diterpenoid quinones, is derived from *Salvia miltiorrhiza*. This compound demonstrates notable pharmacological activities, which include antioxidative and antiapoptotic effects. At present, its primary applications are in the management of ischemic cardiovascular conditions, such as coronary heart disease and angina pectoris.^[22]^ In a study conducted by Chen,^[23]^ the impact of Tan IIA was explored using an atherosclerosis (AS) mouse model, where it was administered via intraperitoneal injection. Findings revealed that Tan IIA possesses the ability to restrict the activation and release of pro-inflammatory agents by influencing the polarization of macrophages, while simultaneously enhancing the activation and secretion of anti-inflammatory factors from M2 macrophages, thus facilitating anti-inflammatory responses. Lyu Yanjie^[24]^ studied the impact of Tan IIA in a rat model of renal interstitial fibrosis, also via intraperitoneal injection. The findings revealed that Tan IIA could down-regulate platelet-derived growth factor-D (PDGF-D), up-regulate heme oxygenase-1 (HO-1) protein expression, and inhibit interstitial cell proliferation and phenotypic transformation, thus contributing to its anti-renal interstitial fibrosis role.

#### 4.2.2. Luteolin

Luteolin is a yellow crystalline compound with a needle-like shape, commonly extracted from various traditional Chinese medicinal herbs, such as wild chrysanthemum and *Prunella vulgaris*.^[25]^ Research indicates that luteolin can suppress the expression of inflammatory mediators and lessen the influx of inflammatory cells via multiple signaling mechanisms. In a study involving mice on a high-fat diet, it was observed that luteolin primarily obstructs the TLR signaling pathway and lowers the plasma levels of cytokines like monocyte chemoattractant protein-1, macrophage inflammatory protein-1β, and plasminogen activator inhibitor-1, thereby alleviating the inflammatory response.^[26]^ Additionally, luteolin significantly diminishes plasma concentrations of sCD163, hinders macrophage activation, and reduces both macrophage infiltration and clustering in the liver and adipose tissues.^[27]^ According to Zhang,^[28]^ luteolin has the ability to curb inflammatory responses and oxidative stress, minimize renal fibrosis, and slow down the advancement of renal damage by repressing the STAT3 pathway in a diabetic mouse model that received a dosage of 50 mg/kg of luteolin.

#### 4.2.3. Ellagic acid

Ellagic acid is a natural polyphenol compound and a derivative of gallic acid. It exhibits various pharmacological effects, including anti-inflammatory, antiviral, and immune-regulatory properties. Studies have demonstrated that ellagic acid can effectively reduce serum levels of TNF-α, NF-κB, IL-1β, IL-6, and COX-2 in rats with lead-induced renal injury, while also improving renal tissue structure.^[29]^ Furthermore, ellagic acid and its derivatives possess the capability to scavenge DPPH free radicals, ABTS free radicals, hydroxyl radicals, and superoxide anion radicals,^[30]^ thereby reducing oxidative stress through the mediation of the Nrf2 pathway.^[31]^ Additionally, ellagic acid significantly decreased serum creatinine, 24-hour microalbumin, and TNF-α levels in diabetic mice. Renal histological evaluations using HE and PAS staining revealed a marked improvement in mesangial proliferation, suggesting that ellagic acid may alleviate renal injury in diabetic mice through its antioxidant effects.^[32]^

#### 4.2.4. Baicalein

Baicalein is a flavonoid component derived from *Scutellaria baicalensis*, known for its anti-inflammatory, antioxidant, immune-regulating, antiviral, antiapoptotic, and antibacterial properties.^[33]^ It has been shown to reduce the inflammatory response associated with pathogenic microorganism infections, inhibit the secretion of various inflammatory mediators, and exhibit both direct antiviral and synergistic antiviral effects. HAN^[34]^ reported that baicalein demonstrated high activity in free radical scavenging assays. Additionally, Zhang^[35]^ conducted cell experiments that revealed baicalein’s ability to significantly inhibit the release of inflammatory factors such as nitric oxide, tumor necrosis factor-alpha (TNF-α), IL-6, and the expression of inducible nitric oxide synthase and cyclooxygenase-2 proteins. Baicalein also exhibited notable scavenging effects on DPPH free radicals and superoxide anions, indicating strong anti-inflammatory and antioxidant activities.

#### 4.2.5. Quercetin

Quercetin, a notable flavonoid compound, is recognized for its various biological functions, which encompass antioxidant, anti-inflammatory, anti-cancer, antibacterial, antiviral, and immune-regulatory activities. According to Zhang,^[36]^ quercetin has the ability to lower the concentrations of inflammatory factors generated by macrophages, including TNF-α, IL-6, and IL-1β, which highlights its anti-inflammatory effects and potential for future therapeutic use in managing inflammatory diseases. In a study by Yuan,^[37]^ it was observed that quercetin can impede neutrophil infiltration, diminish plasma levels of inflammatory cytokines, and enhance the apoptosis of activated neutrophils.

## 5. Conclusion

In this study, DLN was utilized as the reference object to identify the immune core genes associated with RLN, preliminarily elucidating the drug resistance mechanisms of RLN and analyzing potential therapeutic drugs. This approach aims to provide valuable insights for the clinical treatment of RLN patients. However, this study has certain limitations. First, the results are derived from public biological databases and lack direct verification through clinical samples. Secondly, the immune function and mechanisms of RLN require further investigation, which will be the focus of future research.

## Acknowledgments

Thank you to all the authors who have worked hard for this research institute. For your work, we are indispensable. We hope that our cooperation can continue forever and contribute to the development of medical undertakings.

## Author contributions

**Data curation:** Shui Wan, Zhengkai Fan.

**Formal analysis:** Zhongxiong Han.

**Methodology:** Shui Wan.

**Project administration:** Jinbo Pi, Pingping Sun.

**Software:** Kaixiu Wu, Zhongxiong Han.

**Validation:** Yanggen Zuo.

**Visualization:** Li Xiao.

**Writing – original draft:** Bo Shao.

**Writing – review & editing:** Pingping Sun.
